# Comparison of simple and rapid collection methods for measuring salivary IL-6 and PGE_2_ in cats with chronic gingivostomatitis: a pilot study

**DOI:** 10.29374/2527-2179.bjvm012125

**Published:** 2026-06-19

**Authors:** Rita de Cássia Anaya Gutierrez, Luciana Bandini, João Filipe Requicha, Silvia Lourenço, Cristina de Oliveira Massoco Salle Gomes

**Affiliations:** 1 Departamento de Patologia (VPT), Faculdade de Medicina Veterinária e Zootecnia, Universidade de São Paulo (USP), São Paulo, SP, Brazil.; 2 Departamento de Ciências Veterinárias, Universidade de Trás-os-Montes e Alto Douro, Vila Real, Portugal.; 3 Faculdade de Odontologia, Universidade de São Paulo (USP), São Paulo, SP, Brazil.

**Keywords:** feline chronic gingivostomatitis, inflammation, saliva, IL-6, PGE_2_, gengivoestomatite crônica felina, inflamação, saliva, IL-6, PGE_2_

## Abstract

Feline chronic gingivostomatitis (FCGS) is characterized by persistent oral inflammation, leading to severe pain, hyporexia, weight loss, and reduced well-being. Affected cats exhibit altered levels of inflammatory mediators; however, most diagnostic procedures require administration of anesthesia, highlighting the need for alternative monitoring approaches. This prospective exploratory pilot study aimed to compare two simple and rapid saliva collection methods—pipetting and absorbent paper points—used for assessing prostaglandin E_2_ (PGE_2_) and interleukin-6 (IL-6) levels in cats. Saliva samples were obtained from 86 cats that were initially evaluated and classified into three groups: FCGS (n=22), other oral inflammatory conditions (OOIC) (n=27), and controls without oral inflammation (n=37); 25 of the control animals were subsequently excluded from the analysis. Compared with pipetting, absorbent paper points were better tolerated by cats with oral inflammation. In the samples collected by pipetting, the mean IL-6 concentration was higher in the FCGS group (473.10 pg/mL) than in the control group (12.78 pg/mL). A similar pattern was observed in samples collected using absorbent paper points; IL-6 levels were highest in the FCGS group (170.90 pg/mL), followed by the OOIC (6.05 pg/mL) and control (1.50 pg/mL) groups. PGE_2_ levels were also highest in the FCGS group (647.52 pg/mL), followed by the control group (182.13 pg/mL) and the OOIC group (111.40 pg/mL). These findings should be interpreted in light of the pilot design, sample exclusions, and absence of a diagnostic reference standard. Nonetheless, salivary assessment appears to be a feasible and promising noninvasive approach for monitoring oral inflammation in cats with FCGS.

## Resumo

A gengivoestomatite crônica felina (Feline Chronic Gingivostomatitis – FCGS) é caracterizada por inflamação oral persistente, resultando em dor intensa, hyporexia, perda de peso e redução do bem-estar. Gatos acometidos apresentam alterações em mediadores inflamatórios; entretanto, a maioria dos procedimentos diagnósticos requer anestesia, o que evidencia a necessidade de abordagens alternativas para monitoramento da doença. Este estudo prospectivo, piloto e exploratório teve como objetivo comparar dois métodos simples e rápidos de coleta de saliva — pipetagem e pontas de papel absorventes — para a avaliação dos níveis de prostaglandina E_2_ (PGE_2_) e interleucina-6 (IL-6) em gatos. Amostras de saliva foram obtidas de 86 gatos inicialmente avaliados e classificadas em três grupos: FCGS (n=22), outras condições inflamatórias orais (Other Oral Inflammatory Conditions – OOIC; n=27) e controles sem inflamação oral (n=37), dos quais 25 animais controle foram posteriormente excluídos da análise. As pontas de papel absorventes foram melhor toleradas por gatos com inflamação oral. Nas amostras coletadas por pipetagem, os níveis de IL-6 foram mais elevados no grupo FCGS (média de 473,10 pg/mL) em comparação aos controles (12,78 pg/mL). Padrão semelhante foi observado nas amostras coletadas com pontas de papel absorventes, com maiores níveis de IL-6 no grupo FCGS (170,90 pg/mL), seguido por OOIC (6,05 pg/mL) e controles (1,50 pg/mL). Os níveis de PGE_2_ também foram mais elevados no grupo FCGS (647,52 pg/mL), seguidos pelos controles (182,13 pg/mL) e pelo grupo OOIC (111,40 pg/mL). Esses achados devem ser interpretados à luz do delineamento piloto do estudo, das exclusões amostrais e da ausência de um padrão diagnóstico de referência aplicado de forma uniforme. Ainda assim, a avaliação salivar demonstra ser uma abordagem não invasiva, viável e promissora para o monitoramento da inflamação oral em gatos com FCGS.

## Introduction

Feline chronic gingivostomatitis (FCGS) is an immune-mediated disease of unknown etiology and is characterized by chronic oral mucosal inflammation. Mucosal inflammation of the mucosa extends beyond the mucogingival junction and progresses caudally and bilaterally, causing erosion, ulceration, or proliferative changes (Soltero-Rivera et al. 2023). Clinical signs include moderate to severe pain, sialorrhea, hyporexia, anorexia, and weight loss (Jennings et al., 2015; Arzi et al., 2016). Currently, no single effective treatment is available, but dental extractions combined with multimodal medical therapy involving analgesics, anti-inflammatory agents, antibiotics, and immunosuppressants are indicated to reduce clinical signs (Jennings et al., 2015; Lommer, 2013).

Cats with FCGS exhibit significant alterations in inflammatory mediators, including increased levels of IL-4, IL-6, IL-10, IL-12, and IFNγ and decreased levels of IgA, along with a moderate increase in IgM and a significant increase in IgG (Harley et al., 1999; Dolieslager et al., 2013). Peralta et al. (2023) investigated gene expression in the oral mucosa of cats with FCGS and healthy cats and reported an inflammatory profile characterized by the expression of NfkB, JAK/STAT, IL-17, and type I and type II IFN, which is largely influenced by IL-6 (Peralta et al., 2023).

Prostaglandin E_2_ (PGE_2_) is among the most frequently evaluated biomarkers of inflammation in mammals and is correlated with the inflammatory processes associated with orthodontic movement. In that context, PGE2 levels were elevated in the gingival crevicular fluid collected from all cats, with higher levels observed in cats in anestrus or those that had been ovariectomized (Celebi et al., 2013).

Given the disease prevalence of 0.7% to 12%, approximately 30% refractoriness to tooth extraction therapy, and the unclear etiology, numerous studies have focused on FCGS. However, most of these studies have relied on biopsies for monitoring the response to new therapies, procedures that require sedation and/or general anesthesia. Many of these cats are not favorable candidates for anesthesia due to their clinical condition, and the procedure is costly. Therefore, minimally invasive techniques must be investigated for monitoring these patients.

Saliva has been used as a biological sample for various analyses, including cortisol measurement in felines, detection of viruses (e.g., SARS-CoV-2), and the assessment of interleukins in human dentistry, which have proven to be effective and easy to perform (Nguyen-Kim et al., 2022). The development of standardized saliva collection techniques that can be reliably replicated may contribute to routine clinical practice, not only for diagnosis but also for disease monitoring and evaluation of responses to established treatments.

As various diseases, including oral diseases, have been correlated with functional changes involving one or more cytokines and/or inflammation biomarkers, this study aims to characterize the inflammatory profile of cats with chronic gingivostomatitis in comparison with that of cats without the disease by using saliva as a biosample. A minimally invasive procedure that does not require anesthesia or sedation could contribute to the monitoring of responses to new treatments for this disease within the scientific community.

## Materials and methods

### Selection of animals

All animals included in this project were domiciled in São Paulo, Brazil, and its metropolitan region. The study was approved by the institutional ethics committee.

Initially, 86 cats were included in this study. Based on the results of the clinical examination of the oral cavity by using a visual scale, the selected animals were divided into three groups: Group A (grade 0, control group; n = 37) included cats without clinically detectable oral inflammation; saliva was collected during elective sterilization surgery. Of these, 25 animals were excluded due to insufficient volume of saliva. Group B (grades 2–3) comprised cats with other inflammatory conditions (OOIC; n = 27), and Group C (grade 4) included cats diagnosed with FCGS (n = 22).

**Figure 1:**
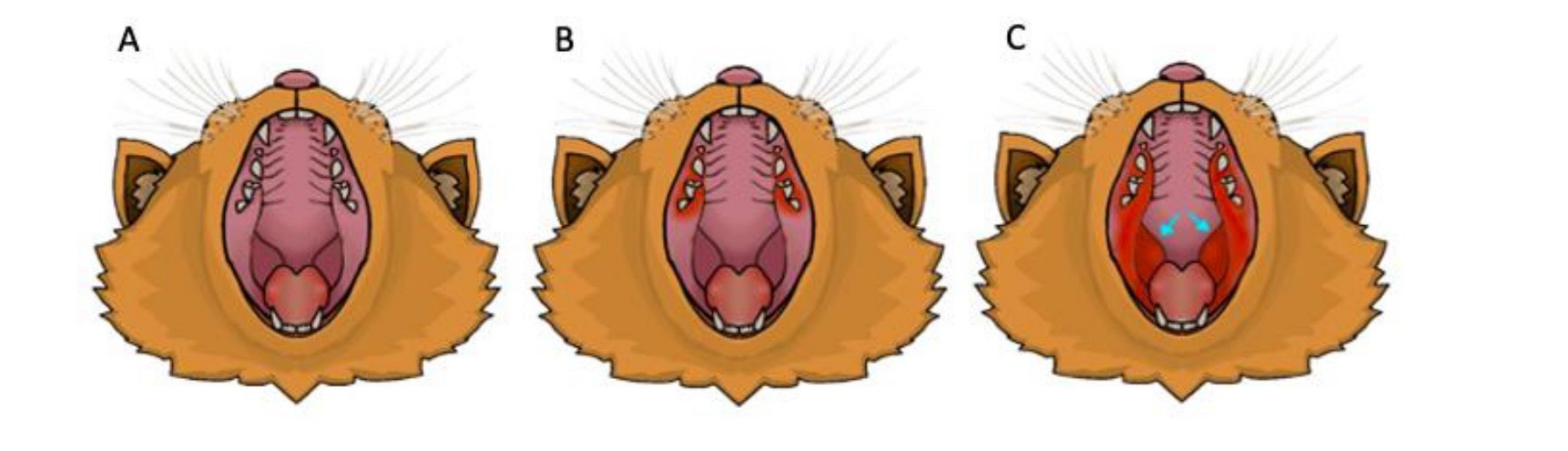
Score classification in oral cavity examination: (A) grade 0, absence of injuries; (B) grade 2–3, moderate to severe gingivitis associated with dental calculus; (C) grade 4 (FCGS), gingivitis (with or without calculus) associated with proliferative and/or ulcerative lesions in the palatoglossal fold and/or mucous membranes and/or tongue (extragingival lesions).

The visual scale used to examine the oral cavity and accordingly establish groups was described by Tenorio et al. (1990) for feline oral lesions. Details regarding comorbidities were addressed separately. The oral health of all cats was evaluated by an experienced veterinary dentist to ensure appropriate classification. The FCGS group included felines with no prior surgical treatment or those refractory to surgical intervention. The classification pattern used in this study is shown in Figure 1.

Illustrations created by the authors using Ibis Paint.

### Histological analysis

Gingival biopsies of patients who underwent dental treatment were collected for histological analysis. Histological examinations were performed on eight felines: six with FCGS and two with periodontal disease. After fixation in 10% neutral buffered formalin for 24 h, the samples were oriented by identifying the oral epithelium and sectioned perpendicularly along the longitudinal diameter. All samples were embedded in paraffin wax. Serial 5-µm sections were cut, stained with hematoxylin and eosin, and examined under an Olympus light microscope. Photomicrographs were obtained using a camera system.

### Study design for sample collection

Two protocols were tested to determine the best method for saliva collection in cats.

**Figure 2:**
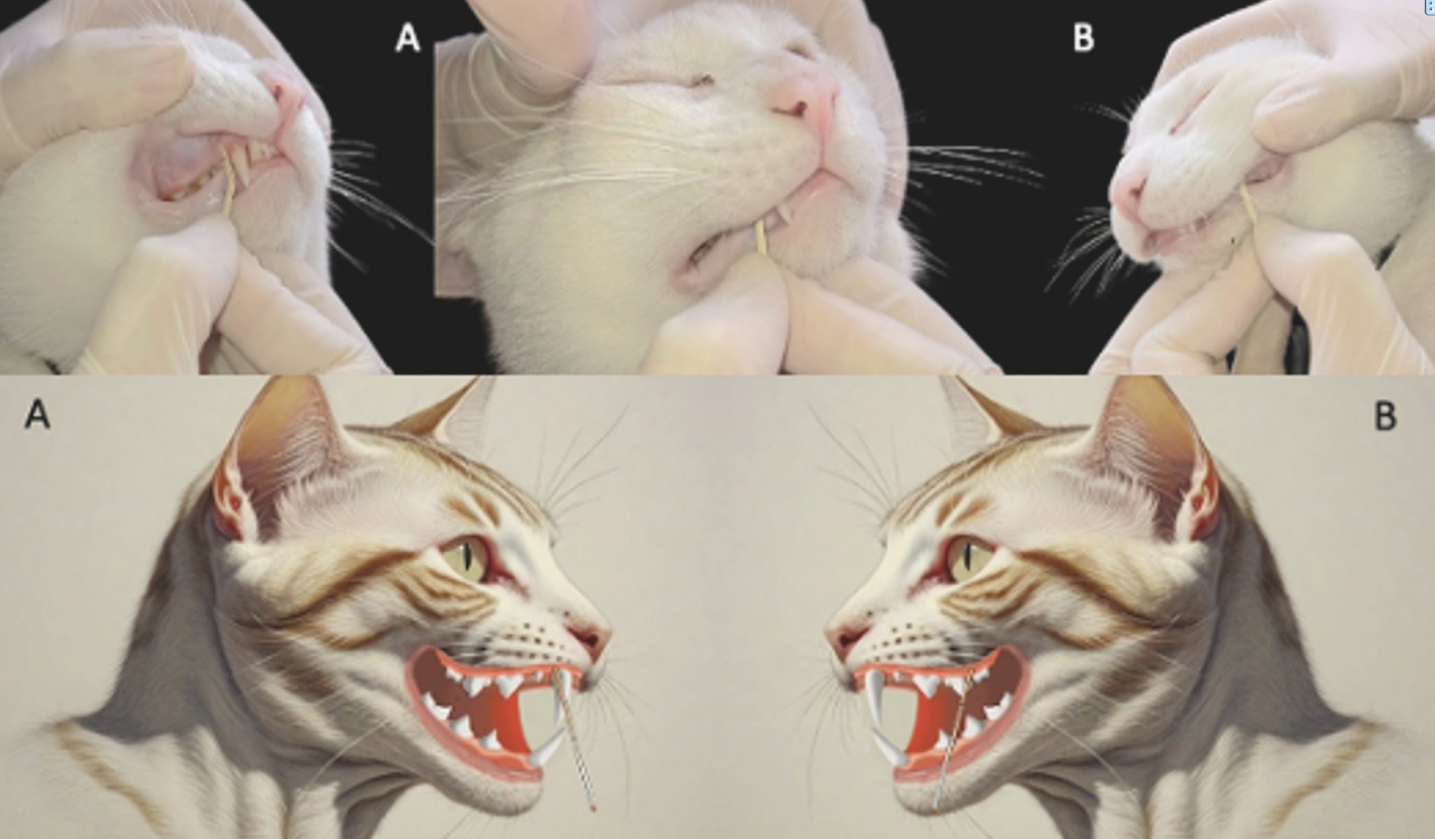
Saliva collection using absorbent paper points: (A) canine region; (B) premolar region.

1. **Protocol I-Pure saliva:** Saliva was collected using a 200-µL pipette tip. Due to the viscosity of the saliva, a small cut was made at the tip to increase the diameter of the opening. Saliva was collected in fractions until a volume of 200 μL was reached, depending on salivary production. However, in some patients, the desired volume could not be collected, and the sample was discarded. The saliva samples were kept at 8 °C, transported to the laboratory, and subsequently frozen at -80 °C.
2. **Protocol II**-**Saliva collection using absorbent paper points**: Five absorbent paper points (size 25) were placed on the gingival mucosa, starting at the canine teeth and subsequently moving to the premolar region (Figure 2A, B). The points remained in place for 60 to 90 seconds until approximately three-quarters of the paper point length was saturated. In total, three collections were performed for each animal. The points were placed in microtubes containing the sample, 200 µL of sterile PBS was added to each sample containing five absorbent paper points (total of 15 paper points per animal), and the samples were then frozen at −80 °C (Figure 2A, B).

Upper image: personal archive (original photograph). Lower image: illustration created by the authors using Ibis Paint.

Saliva samples from Protocol II cats were subjected to two analytical procedures: IL-6 measurement and/or PGE_2_ measurement. Whenever possible, both inflammatory mediators were analyzed; only one mediator was measured for cases in which the sample volume was insufficient

Table 1 shows the number of cats analyzed for each procedure and the number of samples discarded per protocol and/or measurement.

**Table 1:** Samples in each protocol and/or inflammatory mediator measurement and discarded samples.

**Groups (samples)**	**Protocol and inflammatory mediators’ measurement**					**Total**
	**Protocol I – Pure saliva**		**Protocol II – Absorbent paper points**			
	**IL-6**	**IL-6 samples excluded**	**IL6 + PGE_2_**	**Only IL6**	**Only PGE_2_**	
A) Control	5	25	7	-	-	37
B) OOIC	-		22	4	1	27
C) FCGS	7		10	3	2	22

OOIC: other oral inflammatory conditions; FCGS: feline chronic gingivostomatitis.

### ELISA IL-6 and PGE_2_ measurements

ELISAs for feline IL-6 (Quantikine) and PGE_2_ (Cayman) were performed according to the manufacturers' protocols. Samples were thawed and subsequently centrifuged for 5 min at 350 × g. For the initial IL-6 and PGE_2_ assays, samples from Protocol I (pure saliva) were analyzed undiluted and at 1:2 and 1:10 dilutions. For Protocol II (saliva collected using absorbent paper points), samples were analyzed undiluted and at a 1:2 dilution. For both the IL-6 and PGE_2_ analyses, undiluted samples were used for data interpretation.

### Data analysis

The data obtained were statistically analyzed using JASP (version 2023) and GraphPad Prism 6^®^, following verification of data normality using the D’Agostino–Pearson test. Data were considered to be normally distributed (parametric)when p > 0.05**.** The Shapiro–Wilk test was applied for nonparametric data, and statistical significance was set at 5% (p < 0.05).

## Results

**Figure 3:**
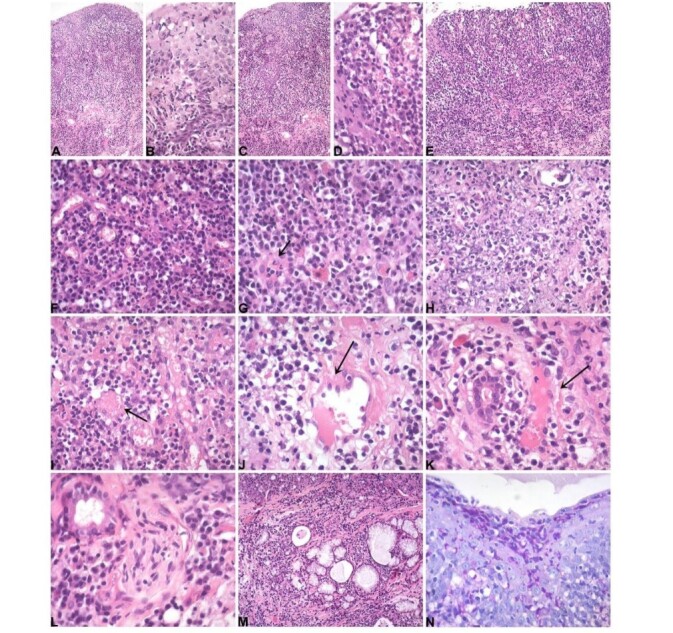
Representative histological images of gingival tissue from cats with FCGS. (A–C) Mucosal epithelium with acanthosis, spongiosis and pseudoepitheliomatous hyperplasia, showing lymphocyte and neutrophil exocytosis. Note (B) the abundant presence of dendritic cells intermingling epithelial keratinocytes (H&E, original magnification ×100, ×400 and ×100, respectively). (D, E) Ulcerated area associated with granulation tissue (H&E, original magnification ×100 and ×400, respectively). (F) Granulation tissue in the lamina propria (H&E, original magnification ×400). (G) Intense inflammatory infiltrate composed of polymorphonuclear neutrophils, lymphocytes and plasmocytes; note the exuberant endothelial edema and necrosis of vessel walls (H&E, original magnification ×400). (H) Necrotic area intermingled with histiocytes and macrophages (H&E, original magnification ×400). (I–K) Necrosis of the blood vessel wall (arrow in I) and the presence of fibrin deposits surrounding blood vessels (leucocytoclastic vasculitis) with hyaline thrombi (arrows in J and K) (H&E, original magnification ×400, ×400 and ×400, respectively). (L) Perineural inflammatory infiltrate (H&E, original magnification ×400). (M) Intense sialadenitis (H&E, original magnification ×100). (N) Presence of neutrophil debris within the upper layers of the epithelium revealed by PAS histochemical staining (original magnification ×400).

Microscopic evaluation of gingival epithelial sections from patients with FCGS revealed intense inflammation, as illustrated in Figure 3AL. The histological evaluation of the gingival biopsies is described in the figure legend.

### Protocol I: Pure saliva

The results of Protocol I (pure saliva) are summarized below; detailed individual animal-level data are provided in Supplementary Table S1.

- **Control group, grade 0 (n = 5)**: The mean age of the animals in the control group was 7 ± 2.86 months. This group consisted of four males and one female; all animals were mixed-breed. The mean IL-6 concentration was 12.78 ± 14.09 pg/mL.
- **OOIC group, grades 2–3 (n = 0)**: No cats from this group were included in Protocol I.
- **FCGS group, grade 4 (n = 7)**: The mean age of the animals in the FCGS group was 6.57 ± 3.74 years. This group consisted of four males and three females; all animals were mixed-breed. None of the animals had undergone surgical treatment, and at the time of sample collection, they were not receiving medication. The mean IL-6 concentration was 473.10 ± 750.7 pg/mL.

**Figure 4:**
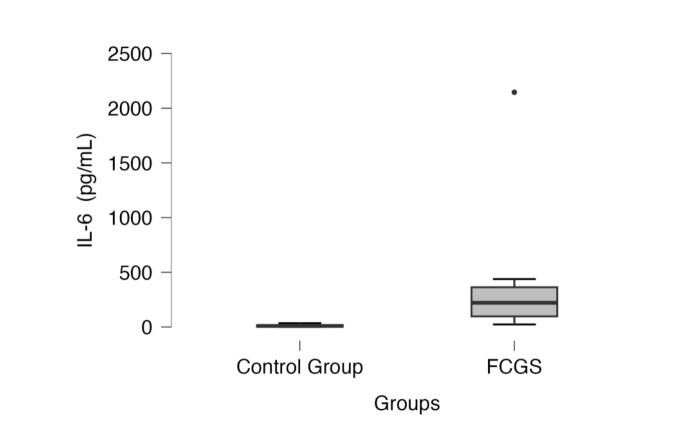
IL-6 concentrations in pure saliva samples (Protocol I) from Groups A and C.

Comparisons between the groups revealed a statistically significant difference (p = 0.005), as illustrated in Figure 4.

### Protocol II: Saliva collection using absorbent paper points and IL-6 measurement

The results of Protocol II, in which saliva was collected using absorbent paper points for IL-6 measurement, are summarized below. Detailed individual animal data are provided in Supplementary Table S2.

- **Control group, grade 0 (n = 7):** The mean age of the animals in this control group was 7.0 ± 3.91 months. This group consisted of three females and four males; all the animals were mixed-breed. One cat from this group presented with feline idiopathic cystitis and urethral obstruction. The mean IL-6 concentration was 1.50 ± 3.99 pg/mL.
- **OOIC group, grades 2–3 (n = 26):** The mean age of the animals in this OOIC group was 6.96 ± 3.73 years. This group consisted of 11 females and 15 males; 23 animals were mixed-breed, two were Maine Coons, and one was a Persian. Six cats from this group presented with moderate periodontal disease (grade 3), one cat was paraplegic, and one cat was anesthetized for plate and screw removal following femoral osteosynthesis. The mean IL-6 concentration was 6.05 ± 15.09 pg/mL.
- **FCGS group, grade 4 (n = 13):** The mean age of the animals in this FCGS group was 7.0 ± 3.29 years. This group consisted of five females and eight males; three cats were Maine Coons and ten were mixed-breed. Two cats from this group were refractory to tooth extraction, one of which was receiving oral probiotic supplements. One cat was under corticosteroid treatment. In three cats, saliva was collected on the day of partial tooth extraction, whereas in ten cats, saliva was collected without sedating the animals. The mean IL-6 concentration was 170.90 ± 324.85 pg/mL.

**Figure 5:**
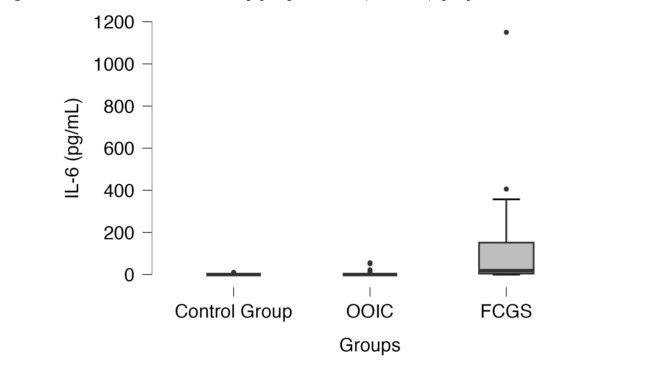
IL-6 concentrations in saliva samples collected from Groups A, B and C by using absorbent paper points (Protocol II).

Comparisons between groups revealed a statistically significant difference (p < 0.001) (Figure 5).

### Protocol II: Saliva collection using absorbent paper points and PGE_2_ measurement

The results of Protocol II, in which saliva was collected using absorbent paper points for PGE_2_ measurement, are summarized below. Detailed individual animal data are provided in Supplementary Table S3.

- **Control group, grade 0 (n = 7):** The mean age of the animals in this control group was 7.5 ± 3.83 months. This group consisted of three females and four males; all the animals were mixed-breed. One cat from this group presented with feline idiopathic cystitis and urethral obstruction. The mean PGE_2_ concentration was 182.13 ± 195.28 pg/mL.
- **OOIC group, grades 2-3 (n = 23)**: The mean age of the animals in this OOIC group was 7.0 ± 3.68 years. This group consisted of 13 females and 10 males; 21 were mixed-breed cats and two Maine Coons. Five animals from this group had moderate periodontal disease (grade 3), one patient was paraplegic, and one patient had saliva collected during plate and screw removal following femoral osteosynthesis. The mean PGE_2_ concentration was 111.40 ± 169.78 pg/mL**.**
- **FCGS group, grade 4 (n = 12)**: The mean age of the animals in this FCGS group was 6.75 ± 3.62 years. This group consisted of five females and seven males; two were Maine Coons and ten were mixed-breed cats. One patient from this group was refractory to full-mouth extraction and was receiving oral probiotic therapy. Two patients in this group received corticosteroid therapy. On the day of partial tooth extraction, saliva was collected from three animals who received no prior medication. The remaining animals had not undergone surgical treatment at the time of collection, and saliva was obtained from them without administering anesthesia. The mean PGE_2_ concentration was 647.52 ± 299.72 pg/mL.

**Figure 6:**
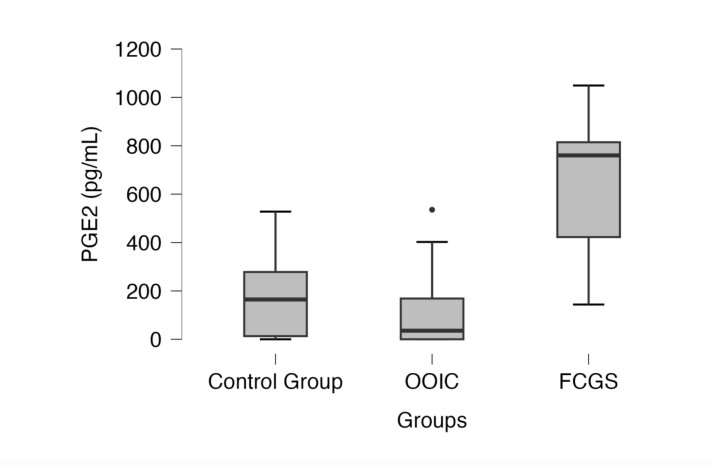
PGE_2_ concentrations in saliva samples collected from Groups A, B and C by using absorbent paper points (Protocol II).

Comparisons between groups revealed a statistically significant difference (p < 0.001) (Figure 6).

## Discussion

Monitoring FCGS using minimally invasive biomarkers complements clinical evaluation and enables the assessment of treatment response. In this study, we compared pure saliva collected using a simple method with saliva collected using absorbent paper points. In a study, saliva was collected from cats by using a pipette tip for measuring allergens, lipocalins, Fel d 1, and Fel s 4 (Kelly et al., 2018). However, the use of absorbent paper points for saliva collection is better tolerated in cats with FCGS, as inflamed oral tissues are highly sensitive. This method reduces patient discomfort during sampling. The absorbent paper points were initially placed on the gingiva in the canine tooth region, which exhibited a lower degree of inflammation. As the points absorbed saliva, they became more malleable and were better tolerated. Subsequently, the points were placed in the premolar region extending toward the palatoglossal region.

Measuring IL-6 and PGE_2_ was feasible using both collection methods. Absorbent paper points were better tolerated by cats with FCGS, although lower IL-6 concentrations were consistently observed across all groups with this than with saliva collected using a pipette tip.

Another study used filter paper to collect saliva from dogs to measure salivary cortisol levels and reported good accuracy with this sampling method (Oyama et al., 2014). In addition, other collection methods that have been described to be used for dogs, including the use of different forms of ophthalmic sponges and cotton (balls, cylinders, pads, and buds), may generate friction on inflamed oral tissues in felines, potentially worsening their pain (Cronin et al., 2003; Dreschel & Granger, 2009; Kotrschal et al., 2009).

Saliva is increasingly recognized as a promising biological sample for various diagnostic and treatment monitoring methods and can also be an excellent tool for assessing inflammation in the oral cavity. In human dentistry, several tests, such as those for detecting hormones, drugs, and toxins, as well as polymerase chain reaction (PCR) for detecting viral and bacterial diseases, use saliva as a sample for analysis. *Helicobacter pylori* was detected in the saliva of patients, showing a strong correlation with gastric biopsy findings, with results obtained from saliva samples being comparable to those obtained from biopsy (Tiwari et al., 2005).

In the field of veterinary medicine, Nickel et al. (2022) compared salivary and serum urea levels using test strips and reported a positive correlation between the results. However, as the test is semiquantitative, it has several limitations, especially at relatively high concentrations. In dogs, saliva has also been used to detect anti-Leishmania antibodies, with good accuracy noted for both IgG2 and IgA anti-Leishmania antibodies (Cantos-Barreda et al., 2017). In that study, saliva samples were collected using Salivette tubes (Sarstedt, Nümbrecht, Germany).

Dodds (2018, 2019) conducted two studies on food intolerance, the first in dogs in 2017 and the second in cats in 2019. They compared serum and salivary levels of allergen-specific IgM and IgA. The results demonstrated that saliva is a reliable alternative in both species when collected using a dental cotton rope.

IgM, IgG, and IgA levels have been investigated in the saliva and serum of patients with FCGS. Immunoglobulin levels were higher in the serum of affected patients. However, IgM and IgG levels were higher in saliva, whereas IgA levels were lower compared with those in the control group (Harley et al., 2003).

During inflammation, phagocytes are activated at the site of tissue injury, followed by the production of cytokines such as tumor necrosis factor (TNF-α), IL-1, and IL-6. This generates the acute phase reaction (APR**)**, which results in the modulation of protein synthesis by leukocytes (Ceciliani et al., 2002). IL-6, like IL-1, has a stimulatory role in the production of acute phase proteins (APP), which can vary by more than 25% relative to basal levels.

Another study identified IL-6 as a key orchestrator of the inflammatory response in FCGS, underscoring the importance of its evaluation. Our results revealed a marked difference in IL-6 levels between sick and healthy felines. Of note, even cats with moderate periodontal disease did not exhibit IL-6 concentrations as high as those observed in cats with FCGS, which is consistent with the findings reported by Peralta et al. (2023). In the present study, sick felines that exhibited lower IL-6 levels were receiving corticosteroid or probiotic therapy, suggesting a modulatory effect of these interventions on systemic inflammation. Additionally, a basal level of inflammatory cytokines is physiologically maintained, as the oral mucosa is continuously exposed to minor insults during normal feeding, reflecting an ongoing immune response to recurrent low-grade bacterial challenges.

Markers of inflammation in the oral cavity have gained increasing prominence in human medicine. Serum and gingival crevicular fluid samples have traditionally been considered the gold standard for evaluating interleukins; however, saliva has received increasing attention as a less invasive and more easily accessible diagnostic medium, demonstrating results comparable to those obtained with plasma and crevicular fluid (Sexton et al., 2011; Jaedicke et al., 2016). The measurement of salivary interleukins has also been successfully applied to evaluate the effectiveness of nystatin mouth rinses in patients with oral candidiasis, yielding favorable outcomes (Aljaffary et al., 2013).

Recently, salivary cytokines have been investigated in felines with tooth resorption lesions. Among affected cats, subgroups were identified based on oral microbiota diversity, with individuals exhibiting lower microbial diversity presenting higher concentrations of inflammatory interleukins (IL-1β and IL-12p40) and chemokines (IL-8, RANTES, and KC) in addition to TNF-α. This inflammatory profile indicates an active and persistent inflammatory state (Thomas et al., 2024).

Six cats from the FCGS group underwent gingival biopsy; one of these cats, which presented with the most severe form of the disease, additionally underwent a tongue biopsy. Histopathological evaluation revealed a predominance of polymorphonuclear cells, plasma cells, and eosinophils, along with a high density of dendritic cells. In the tongue tissue, an initial suspicion of fungal involvement was raised; however, this hypothesis was ruled out following periodic acid–Schiff (PAS) staining.

A similar pattern was observed for PGE_2_, which remained at higher levels in sick patients. Among cats without FCGS, elevated PGE_2_ concentrations (>300 pg/mL) were detected in six individuals: one cat diagnosed with feline idiopathic cystitis and urethral obstruction, which also exhibited elevated IL-6 levels; one cat anesthetized for plate and screw removal that showed a marked inflammatory process at the implant site accompanied by functional impairment of the affected pelvic limb; three cats with moderate periodontal disease; and one clinically healthy cat. Among sick felines, the lowest PGE_2_ levels were observed in two patients: one was receiving corticosteroid therapy, and the other was not under medication and exhibited lower IL-6 levels.

## Conclusions

In conclusion, salivary analysis appears to be a feasible and promising noninvasive approach for monitoring oral inflammation in cats with chronic gingivostomatitis. Nevertheless, these findings should be interpreted with caution, given the pilot and exploratory nature of the study, the unbalanced sample distribution, and the absence of a uniformly applied diagnostic reference standard across all study groups.

## Supplementary Material

Supplementary material accompanies this paper.

Supplementary Table S1 Individual IL-6 salivary concentrations obtained by Protocol I (pure saliva) in cats from groups A (control) and C (FCGS).

Supplementary Table S2 Individual IL-6 salivary concentrations obtained by Protocol II (absorbent paper points) in cats from groups A (control), B (OOIC), and C (FCGS).

Supplementary Table S3 Individual PGE_2_ salivary concentrations obtained by Protocol II (absorbent paper points) in cats from groups A (control), B (OOIC), and C (FCGS).

This material is available as part of the online article from https://doi.org/10.29374/2527-2179.bjvm012125
