# Supplementary material for: Comparison of simple and rapid collection methods for measuring salivary IL-6 and PGE2 in cats with chronic gingivostomatitis: a pilot study
Source: Braz J Vet Med. 2026 Jun 19;48:e012125. doi: 10.29374/2527-2179.bjvm012125 (PMC13281992; doi:10.29374/2527-2179.bjvm012125)
Supplement: Supplementary Table S1 [file bjvm-48-e012125-suppl.pdf]

Supplementary Table S1. Individual IL-6 salivary concentrations obtained by Protocol I (pure saliva) in cats from groups A (control) and C (FCGS).

ID	Age	Sex	Breed	Group	IL-6 (pg/mL)
5	5 months	M	MB	A) Control	0
12	6 months	M	MB	A) Control	35.00
13	6 months	M	MB	A) Control	9.50
14	6 months	M	MB	A) Control	16.80
20	12 months	F	MB	A) Control	2.41
1	3 years	F	MB	FCGS	71.40
18	4 years	F	MB	FCGS	23.16
31	11 years	M	MB	FCGS	2145.50
32	2 years	M	MB	FCGS	289.50
46	10 years	M	MB	FCGS	437.96
47	6 years	M	MB	FCGS	122.91
48	10 years	F	MB	FCGS	221.30

MB (mixed breed).

Supplementary Table S2. Individual IL-6 salivary concentrations obtained by Protocol II (absorbent paper points) in cats from groups A (control), B (OOIC), and C (FCGS).

ID	Age (years)	Breed	Sex	Group	IL-6 (pg/mL)
50	5	MB	F	B) OOIC	0
51	1	MB	M	A) Control[1]	0
52	0.4	MB	F	A) Control	10.56
53	0,4	MB	F	A) Control	0
54	0,6	MB	M	A) Control	0
55	5	MB	M	B) OOIC	0
56	6	MB	M	B) OOIC	0
57	7	Maine coon	M	B) OOIC	0
58	16	MB	F	B) OOIC	0
59	5	MB	M	B) OOIC	15.74
60	3	MB	M	B) OOIC	0
61	1	MB	F	A) Control	0
62	4	MB	M	B) OOIC [2]	12.34
63	2	MB	F	B) OOIC [3]	0
64	6	MB	F	B) OOIC [4]	0
65	12	MB	M	B) OOIC [2]	55.94
66	14	Persian	F	B) OOIC [2]	0
67	5	MB	M	B) OOIC	0
68	15	MB	M	B) OOIC	0
69	10	MB	M	B) OOIC [2]	51.30
70	4	MB	F	B) OOIC [2]	0
71	6	MB	F	B) OOIC	22.16
72	6	MB	F	B) OOIC	0
73	12	Maine coon	M	B) OOIC	0
74	0,5	MB	M	A) Control	0
75	7	MB	F	B) OOIC	0
76	3	MB	F	B) OOIC	0
77	7	MB	F	B) OOIC	0
78	0,7	MB	M	A) Control	0
79	4	MB	F	B) OOIC	0
80	4	MB	F	B) OOIC	0
81	8	MB	F	B) OOIC [2]	0
82	10	MB	F	B) OOIC [2]	0
33	6	MB	M	FCGS[5]	1150.00

34	5	MB	M	FCGS	13.13
35	3	MB	M	FCGS	405.91
37	7	Maine coon	F	FCGS[6]	4.46
38	3	Maine coon	F	FCGS[6]	8.60
39	10	MB	M	FCGS	18.52
40	8	MB	M	FCGS	4.04
41	13	MB	F	FCGS	84.99
42	5	MB	F	FCGS	19.64
36	7	Maine coon	M	FCGS[6]	3.67
43	5	MB	F	FCGS	151.59
49	13	MB	M	FCGS[5,7]	0
83	9	MB	M	FCGS	357.25

1 patient with urethral obstruction, 2 moderate periodontal disease (grade 2-3), 3 osteosynthesis, 4 paraplegic patient, 5 refractory to partial tooth extraction, 6 corticosteroid therapy, 7 oral probiotic administration.

Supplementary Table S3. Individual PGE_2 salivary concentrations obtained by Protocol II (absorbent paper points) in cats from groups A (control), B (OOIC), and C (FCGS).

ID	Age	Breed	Sex	Group	PGE_2 (pg/mL)
50	5	MB	F	B) OOIC	0
51	1	MB	M	A) Control [1]	527.76
52	0,4	MB	F	A) Control	287.40
53	0,4	MB	F	A) Control	0
54	0,6	MB	F	A) Control	8.94
55	5	MB	M	B) OOIC	35.90
56	6	MB	M	B) OOIC	0
57	7	Maine coon	M	B) OOIC	0
58	16	MB	F	B) OOIC	38.99
60	3	MB	M	B) OOIC	330.00
61	1	MB	F	A) Control	17.09
62	4	MB	M	B) OOIC [2]	36,79
63	2	MB	F	B) OOIC [3]	388.75
67	5	MB	M	B) OOIC	22.20
68	15	MB	M	B) OOIC	0
69	10	MB	M	B) OOIC [2]	0
70	4	MB	F	B) OOIC [2]	0
71	6	MB	F	B) OOIC	0
72	6	MB	F	B) OOIC [2]	339.66
73	12	Maine coon	M	B) OOIC [2]	292.63
74	0,5	MB	M	A) Control	269.12
75	7	MB	F	B) OOIC	35.55
76	3	MB	F	B) OOIC	0
77	7	MB	F	B) OOIC	39.55
78	0,7	MB	M	A) Control	164.61
79	4	MB	F	B) OOIC	44.51
80	4	MB	F	B) OOIC	0
84	8	MB	M	B) OOIC	19.88
81	8	MB	F	B) OOIC [2]	402.36
82	10	MB	F	B) OOIC [2]	535.60
34	5	MB	M	FCGS	190.74
35	3	MB	M	FCGS	812.54
36	7	Maine coon	M	FCGS[6]	143.71
37	4	Maine coon	F	FCGS[6]	444.16
39	10	MB	M	FCGS	616.59
41	13	MB	F	FCGS	781.18
42	5	MB	F	FCGS	809.92
43	4	MB	F	FCGS	1005.30

49	13	MB	M	FCGS[5,7]	739.38
83	9	MB	M	FCGS	820,37
85	4	MB	M	FCGS	>1049.20
86	4	MB	F	FCGS	357.24

1 patient with urethral obstruction, 2 moderate periodontal disease (grade 2-3), 3 osteosynthesis, 5 refractory to partial tooth extraction, 6 corticosteroid therapy, 7 oral probiotic administration
